# Autophagy Impairment in Retinal Ganglion Cells Following Hypoglycemia in Mice

**DOI:** 10.3390/cells14221774

**Published:** 2025-11-12

**Authors:** Daria Fresia, Enrica Cannizzaro, Angelica Borgo, Marc Schwab, Raphaël Roduit

**Affiliations:** Macular Degeneration and Diabetic Retinopathy Group, Department of Ophthalmology, University of Lausanne, Jules-Gonin Eye Hospital, Fondation Asile des Aveugles, Avenue de France 15, CH-1001 Lausanne, Switzerlandangelica.borgo@fa2.ch (A.B.);

**Keywords:** apoptosis, diabetic retinopathy, hypoglycemia, lysosomal fusion defect, RNA-Seq, laser capture microdissection

## Abstract

**Highlights:**

**What are the main findings?**

- The formation of autolysosomes is increased by hypoglycemia both in living tissue and in retinal ganglion cells (RGCs).
- Hypoglycemia induces a defect in the autophagosome/lysosomes fusion process in isolated RGC, removing the protective effect of autophagy.

**What is the implication of the main finding?**

- Modulating the autophagy process may help to prevent complications associated with diabetic retinopathy (DR).

**Abstract:**

(1) Background: Diabetic retinopathy (DR), caused by hypo- and hyperglycaemia, is the leading cause of blindness. Hypoglycemia induces endoplasmic reticulum stress and retinal cell death in mice, and low-glucose conditions induce macroautophagy/autophagy defects in 661W photoreceptor cells and retinal explants. Very few studies have analyzed the effect of hypoglycemia on retinal autophagy, so we decided to fill this gap. (2) Methods: We use C57BL/6 and GFP-LC3 mice and isolated retinal ganglion cells (RGCs) from both mouse models to study the autophagy process. (3) Results: Intraocular injection of rapamycin and 5 h hypoglycemia showed an increase in autophagosomes formation, specifically in the RGCs. Isolated GFP-LC3 RGCs showed an increase in autophagosome formation under low-glucose conditions. In contrast, infection of isolated C57BL/6 RGCs with the RFP-GFP-LC3 lentivirus revealed a defect in autophagosome/lysosome fusion under these conditions. (4) Conclusions: This study showed that 5 h hypoglycemia induces autophagosomes formation in mouse RGCs; however, a defect in the fusion process inhibits the protective effect of autophagy. Therefore, modulating both autophagic and apoptotic pathways might be important to avoid complications associated with DR.

## 1. Introduction

Glucose is one of the retina’s most vital metabolic substrates. Patients with diabetes should maintain normoglycemia to avoid side effects such as cardiovascular disease, nephropathy, neuropathy, and retinopathy. Diabetic retinopathy (DR) is the leading cause of blindness among working-age adults worldwide. Substantial evidence suggests that both hyper- and hypoglycemia are significant causes of DR. Indeed, DR is fundamentally triggered by chronic hyperglycemia, but extensive research indicates that episodes of hypoglycaemia, experienced by many diabetic patients, also play a specific and harmful role in retinal pathology. These episodes are often linked to acute cellular stress and cell death, which, according to descriptions, occur before the onset of DR itself, suggesting that retinal cell degeneration is the first clinical sign of DR, whereas microvascular changes may occur later [1,2]. Moreover, a very recent publication supports the hypothesis that hypoglycemia causes inner blood–retinal barrier breakdown (iBRB), causing retinal vascular leakage [3]. Over 10 years ago, we showed that 5 h hypoglycemia induces retinal cell death in vivo via caspase 3 activation and glutathione downregulation [4]. More recently, we found that hypoglycemia induces endoplasmic reticulum (ER) stress in mice, without glutathione playing a role [5]. Other research groups have identified significant associations between hypoglycemia and diabetic eye disease [6] or visual loss [7]. We have also shown that low-glucose conditions in 661W cells induced apoptosis via the B-cell lymphoma 2/protein Bcl-2-associated X pathway. Low glucose levels also induced autophagy as a protective response via the AMP-activated protein kinase target of the rapamycin pathway. However, we showed that low-glucose conditions promoted autophagosome formation, but a defect in autolysosome (AL) formation led to apoptosis [8].

Autophagy is an essential cellular process for maintaining cellular homeostasis under both physiological and pathological conditions. Autophagy is classified as macroautophagy (often referred to simply as autophagy), chaperone-mediated autophagy, and microautophagy (for review, see [9]). The main step in autophagy is autophagosome formation, followed by a rapid fusion with lysosomes to generate AL, whose contents are degraded by lysosomal enzymes [10]; impairment of fusion can lead to autophagosome accumulation [8]. Autophagy acts as a quality control mechanism playing a key role in retinal development [11] and ocular pathologies, including DR [12,13,14,15,16]. It plays a protective role by degrading mitochondria affected by reactive oxygen species, as shown with a thioredoxin-interacting protein in DR [17]. Autophagy promotes color vision under starvation conditions [18], but it is essential for the survival of retinal ganglion cells (RGCs) [19,20].

The thickness of the RGC layer decreases in patients with DR [21]; such RGC losses are even detectable in patients with diabetes but without DR [22]. Autophagy activation has been demonstrated in different models, including optic nerve transection-induced damage [23], a chronic ocular hypertension rat model [20], and after chronic elevation in intraocular pressure [24]. Although there is strong evidence that autophagy plays a neuroprotective role in damaged RGCs, several studies have reported the opposite effect (for review, see [15]); hence, a complete study of autophagy is important for each model analyzed.

In this study, we investigated the effect of 5 h hypoglycemia on autophagy, using GFP-LC3 and C57BL/6 mice, to highlight the affected retinal cells and the mechanisms underlying this protective pathway. The study objective was to assess the effect of hypoglycemia on RGCs and thereby elucidate the mechanism of autophagy.

## 2. Materials and Methods

### 2.1. Mouse Lines

C57BL/6 and heterozygote GFP-LC3^tg/+^ mice [25], were used as models to study autophagy ex vivo and in vivo. The male and female mice, aged 2 months, were maintained on a 12-h light/12-h dark cycle with unlimited access to food and water before each experiment.

GFP-LC3^tg/+^ mice were intravitreally injected with 2 mL (2.5 mg/mL) of rapamycin (Merck & Cie, Buchs, Switzerland, 553211); 4 h after treatment, their whole eyes were isolated, fixed in 4% paraformaldehyde for 45 min, incubated in a sucrose gradient during 6 h and then embedded in yazzulla (a mixture of 30% egg albumin and 3% gelatin in PBS). The eyes were subsequently cut and analyzed for GFP fluorescence and/or used for colocalization experiments.

Two-month-old female GFP-LC3^tg/+^ mice were also subjected to a hyperinsulinemic clamp, as described earlier [4]. Briefly, an indwelling catheter (Merck & Cie, Darmstadt, Germany) was inserted into the femoral veins of isoflurane-anaesthetized mice, which were allowed to recover for 14 days. After a 5-h fasting period, awake and freely moving mice were subjected to 5 h of either a hyperinsulinemic/hypoglycemic clamp (Hypo) or hyperinsulinemic/euglycemic clamp (Eugly). The mice were sacrificed 4 h after clamp, and their eyes were collected to be fixed and embedded in yazzulla, cut and stained for GFP fluorescence analysis, and/or used for colocalization experiments. For RNA-Seq analysis, 2-month-old female C57BL/6 mice were subjected to a hyperinsulinemic clamp, and their eyes were isolated 4 h later. The eyes were then incubated in a sucrose gradient (5–25%) successively for 30 min at 4 °C; finally, the eyes were incubated in 25% sucrose and OCT (2:1 ratio) for 1 h at 4 °C and mounted in this mixture to be cut.

### 2.2. RGCs Isolation

A simple two-step “panning” protocol was used to isolate mouse RGCs [26]. Briefly, retinas from 0–3 days postnatal C57BL/6 and GFP-LC3^tg/+^ mice (male and female) were isolated and digested with papain. Then, drop-by-drop continuous mechanical dissociation of cells was performed to obtain isolated cells. Macrophages were depleted with anti-CD11b/c (BD Biosciences, Allschwil, Switzerland, 554859)-conjugated CELLection™ Dynabeads™ (Thermo Fisher, Ecublens, Switzerland, 11531D). After collecting the beads using a magnet, suspended cells were directly incubated for 45–50 min at 25 °C on immunopanning plates treated with goat anti-mouse IgM (Jackson ImmunoResearch, Cambridgeshire, UK, 115-005-020) and mouse anti-CD90 (Bio-Rad Laboratories AG, Cressier, Switzerland, MCA02R). After multiple washing steps, RGCs were detached from the plate through incubation with trypsin, counted, and seeded either on 96-well plates or chamber slides (Thermo Fisher, 177380) to be cultured for 48 h in RGC media composed of Neurobasal Medium (Thermo Fisher, 21103049) supplemented with insulin (Merck & Cie, I0516), sodium pyruvate (Thermo Fisher, 11360070), Penicillin–Streptomycin (Thermo Fisher, 15140122), triiodo-thyronine (Merck & Cie, T-6397), L-glutamine (Thermo Fisher, 25030081), N-acetyl cysteine (Merck & Cie, A-8199), forskolin (Merck & Cie, F-3917), and N21-MAX Media supplement (Bio-Techne R&D systems, Zug, Switzerland, AR008) and SATO buffer composed of transferrin (Merck & Cie, T-1147), BSA (Merck & Cie, A-8806), progesterone (Merck & Cie, P-8783), putrescine (Merck & Cie, P-5780), and sodium selenite (Merck & Cie, S-5261).

### 2.3. Cell Culture Conditions

Retinal explants were isolated from 15-day-old mice and cultured on a nitrocellulose filter (Merck & Cie, PIHA03050) in R-16 complete medium [27] for 24 h; then, they were incubated for 48 h in glucose-free Dulbecco’s modified Eagle’s medium supplemented with different concentrations of glucose. C57BL/6 retinal explants were first infected with the mRFP-GFP-LC3 lentivirus and treated with doxycycline, as described earlier [8], prior to culturing as described above. GFP-LC3^tg/+^ retinal explants were cultured at different glucose concentrations in the presence or absence of one of the autophagic modulators, rapamycin (Merck & Cie, 553211) and chloroquine (Merck & Cie, 1118000). The explants were then collected, fixed in 4% paraformaldehyde for 20 min, and embedded in yazzulla after incubation in a sucrose gradient. They were subsequently cut and analyzed for fluorescence.

Isolated RGCs were cultured for 48 h in fresh RGC medium supplemented with brain-derived (Merck & Cie, SRP3014-100G), ciliary (Merck & Cie, C3710-100G), and glial cell line-derived (Merck & Cie, G1777-100G) neurotrophic factors prior to culturing in free Dulbecco’s modified Eagle’s medium supplemented with different glucose concentrations for 48 h. RGCs isolated from C57BL/6 mice were infected with the mRFP-GFP-LC3 lentivirus, treated with doxycycline [8], and then cultured at different glucose concentrations.

### 2.4. Western Blotting Analysis

Protein lysates from isolated RGCs cultured at different glucose concentrations were electrically transferred to PVDF membranes and incubated with the following primary antibodies: rabbit anti-p62/SQSTM1 (Merck & Cie, P0067; 1:5000 dilution) and mouse anti-α-tubulin (Merck & Cie, T6074; 1:1000 dilution) for normalization. IrDye goat anti-rabbit (LI-COR Biotechnology, Bad Homburg vor der Höhe, Germany, 926-32211) or mouse (LI-COR Biotechnology, 926-68070) secondary antibodies were used to detect protein expression with the LI-COR Odyssey DLx imaging system (LI-COR Biotechnology).

### 2.5. Immunostaining

For GFP fluorescence and/or colocalization experiments, whole eyes cut into 8-micron sections were first pre-treated for 10 min in 0.5% Triton X and then blocked for 1 h with 5–10% normal goat serum (Agilent Technologies, Basel, Switzerland, X090710-8), 1% BSA, and 0.1% Triton X-100 (Merck & Cie, X100) in PBS. The sections were then incubated with the primary antibodies, anti-GFAP (Agilent Technologies, Z033401-2; 1:500 dilution), anti-GS (Merck & Cie, G2781; 1:5000 dilution), and anti-RBPMS (Merck & Cie, ABN1362: 1:200 dilution), for the overnight at 4 °C, washed, and incubated with Alexa Fluor 633 secondary antibody (Thermo Fisher, A21072; 1:2000 dilution) for 1 h. After washing, the slides were stained with DAPI for 10 min and mounted. A Leica microscope was used for the analysis.

### 2.6. LCM and RNA-Seq Analysis

Three eyes each from Eugly and Hypo mice were used for LCM. First, retinal sections were placed on Zeiss membrane slides (MembraneSlide 1.0 PET, 415190-9051-000), irradiated with UV light at 254 nm for 30 min, and coated with 0.1% w/v poly-lysine (Sigma Aldrich, St. Louis, MI, USA, P8920). Each retinal slide (10 mm) was first incubated for 30 s in 70% EtOH and then stained with cresyl violet solution for 2 min; the sections were then dehydrated using successive cold EtOH baths (30 s each at 70%, 95%, and 100%) and observed under a microscope. Isolated tissues were transferred to 100 mL of lysis buffer (RNAqueous™-Micro Total RNA Isolation Kit, Thermo Fisher, AM1931) and stored at −80 °C before processing for mRNA preparation. Six retinal mouse samples (isolated RNA)—3 Hypo and 3 Eugly—were evaluated for RNA quality and subjected to RNA-Seq analysis.

Purity-filtered reads were modified by adapters and quality-trimmed with cutadapt (v1.8) [28]. Reads matching ribosomal RNA sequences were removed with fastq_screen (v0.11.1). The remaining reads were further filtered for low complexity with reaper (v15-065) [29]. Reads were aligned against the Mus musculus GRCm38.92 genome using STAR (v2.5.3a) [30]. The number of read counts per gene locus was summarised with htseq-count (v0.9.1) [31] using Mus musculus GRCm38.92 gene annotation. The quality of the RNA-Seq data alignment was assessed using RSeQC (v2.3.7) [32], and isoform abundance was estimated using RSEM (v1.2.31) [33].

### 2.7. Pathway Analysis (Gene Set Enrichment Analysis)

Differential expression results from the RNA-seq comparison of hypoglycemic versus euglycemic ganglion cell layer (GCL) samples were analyzed using Gene Set Enrichment Analysis (GSEA; Broad Institute, version 4.3.3) [34,35]. Genes were supplied to GSEA as a ranked list derived from the differential expression analysis. Gene Ontology (GO) Biological Process autophagy-related gene sets from the Molecular Signatures Database (MSigDB) were used for enrichment testing [36]. GSEA was run with default settings, restricting gene-set size to a minimum of 15 and a maximum of 500 genes. To visualize expression patterns of genes contained in the autophagy-related gene sets, we generated heatmaps in R using the pheatmap package.

### 2.8. Statistical Analysis

All results were expressed as mean ± SEM of the indicated number of experiments. Data were statistically analyzed using Prism 6.0 (GraphPad software, Boston, MA, USA). Each group of data was first tested for distribution normality using the Shapiro–Wilk test. In case of normal distribution, Welch’s ANOVA test (one-way ANOVA with unequal variances), followed by a post hoc Tukey–Kramer test, was performed to compare the different treatments. When the distribution was not normal, the Kruskal–Wallis test (non-parametric analogue of one-way ANOVA) was performed to compare the different treatments. The threshold for statistical significance was set at a *p*-value of <0.05. Statistical significance was calculated using Student’s t-test. For RNA-Seq analysis, statistical analysis was performed for genes in R (v4.0.3 [2020-10-10]). Genes with zero counts in all samples as well as non-protein-coding transcripts were pre-filtered out. Normalization and differential expression analysis were performed using DESeq2 (v1.28.1) [37].

## 3. Results

### 3.1. Low-Glucose Conditions Induced Autophagy Both Ex Vivo and In Vivo

**Figure 1:**
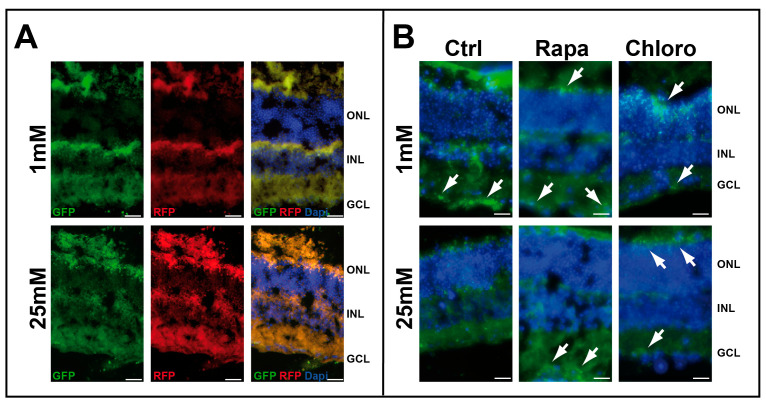
Low glucose concentration induced autophagy. (**A**) Whole-mount retinas of 2-month-old C57BL/6 mice infected with mRFP-GFP-LC3 lentivirus and cultured with 1 and 25 mM glucose. (**B**) Whole-mount retinas of 2-month-old GFP-LC3*^tg/+^* mice cultured with 1 and 25 mM glucose in the presence or absence of autophagic modulators, rapamycin (Rapa) or chloroquine (Chloro). GFP fluorescence (white arrows) is characteristic of autophagosome formation. The images are representative of three different experiments. Scale bar 20 mm.

We initially tested the effect of low-glucose conditions on retinal explants (Figure S1). As we previously demonstrated by Western blot analysis [8] that LC3-II expression increases in retinal explants cultured at low glucose, we decided to test the fusion process using the mRFP-GFP-LC3 chimeric protein. Infection of whole-mount retinas isolated from C57BL/6 mice, using a lentiviral vector expressing mRFP-GFP-LC3 chimeric protein, resulted in strong RFP staining in the presence of both 1 and 25 mM glucose. However, GFP staining was greater under low-glucose conditions compared with high glucose, suggesting abnormal fusion of autophagosomes with lysosomes at low glucose (Figure 1A). Treatment of whole-mount retinas isolated from green fluorescent protein (GFP)-LC3 transgenic (GFP-LC3*^tg/+^*) mice with autophagic modulators (chloroquine or rapamycin) also indicated autophagosome formation at both high (25 mM) and low (1 mM) glucose concentrations. Notably, autophagosomes accumulated at 1 mM glucose (GFP fluorescence), whereas no similar fluorescence was observed at 25 mM glucose in the absence of any autophagic modulators (Figure 1B).

**Figure 2:**
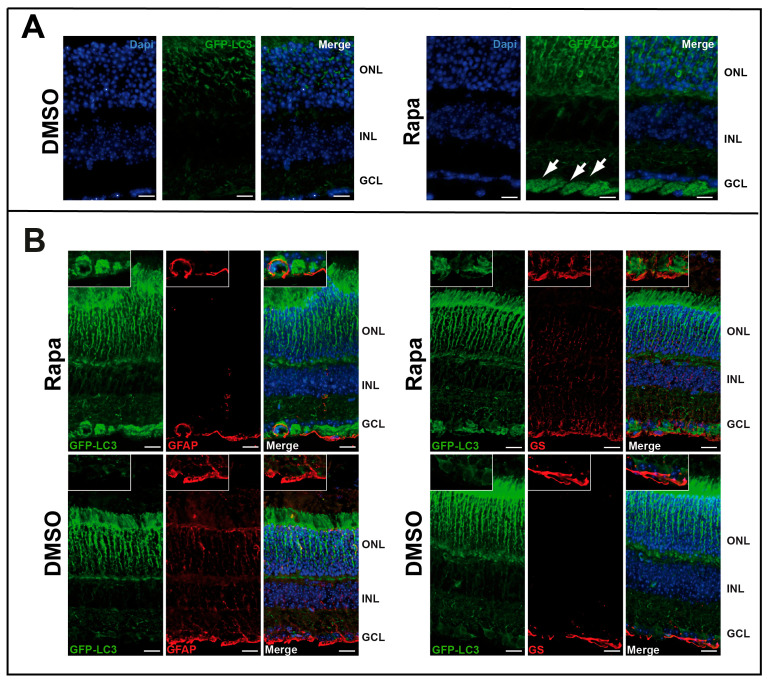
Rapamycin induced in vivo autophagy in GCL. (**A**) GFP fluorescence of retinal sections from GFP-LC3^tg/+^ mice injected intraocularly with DMSO or rapamycin (Rapa). GFP-LC3 accumulation, characteristic of an increase in autophagosomes, is visible in GCL (white arrows). (**B**) Same sections are immuno-stained for glial fibrillary acidic protein (GFAP) or glutamine synthetase (GS) which are both glial markers (the retinal astrocytes and Müller glia, respectively) and associate with GCL and for GFP-LC3. We showed a colocalisation GFP-LC3 with both markers. The images are representative of three different experiments. Scale bar 20 mm.

We then tested the effect of rapamycin in vivo by injecting the drug intraocularly into GFP-LC3*^tg/+^* mice (Figure S1). GFP clearly concentrated in the ganglion cell layer (GCL), demonstrating autophagosome accumulation in this part of the retina (Figure 2A). This result also suggests that the GFP-LC3*^tg/+^* model is suitable to study autophagy in the retina. Immunostaining with glial fibrillary acidic protein (GFAP) or glutamine synthetase (GS) showed that these two proteins colocalized with GFP in some ganglion cells, but not exclusively (Figure 2B); some cells exhibited autophagosome formation but were negative for both GFAP and GS.

**Figure 3:**
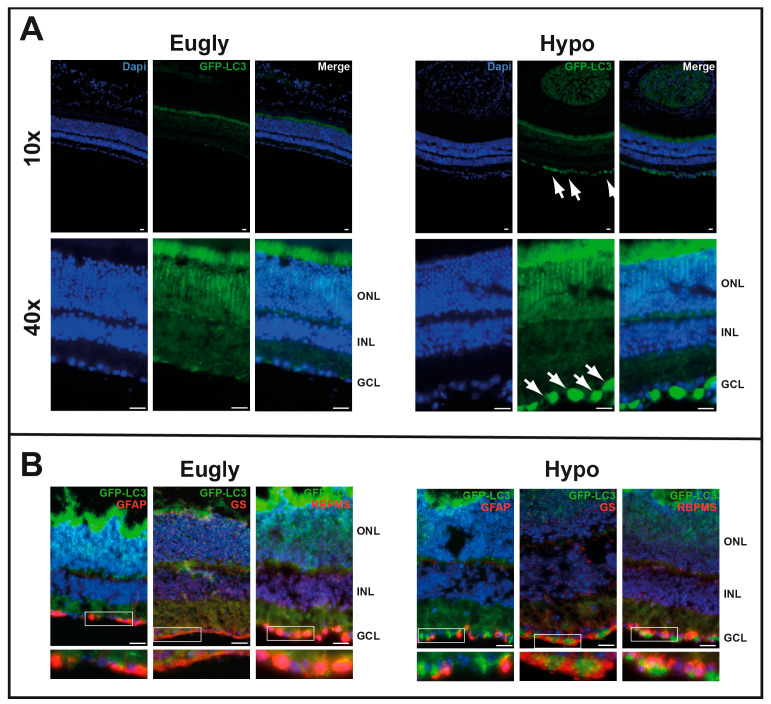
Five-hour hypoglycemia induces in vivo autophagy in GCL. (**A**) A hyperinsulinemic/hypoglycemic clamp (Hypo) was applied to 2-month-old GFP-LC3^tg/+^ mice for 5 h; a hyperinsulinemic/euglycemic clamp (Eugly) was used as control. GFP-LC3 accumulation (GFP fluorescence), characteristic of an increase in autophagosomes, is visible in GCL (white arrows). (**B**) Same sections immuno-stained for GFAP, GS, or RNA-binding protein with multiple splicing (RBPMS), showing GFP-LC3 colocalisation with all three GCL markers. The images are representative of three different experiments. Scale bar 20 mm.

We then tested the impact of 5 h hypoglycemia on autophagosome formation using GFP-LC3*^tg/+^* mice (Figure S1). Mice hypoglycemic for 5 h (Figure 3A) and those injected with rapamycin (Figure 2A) exhibited similar patterns of GFP staining on the GCL; no GFP staining was observed in the control group of mice (Eugly). Both GFAP and GS were found to colocalize with GFP in some ganglion cells but not in others (Figure 3B); GFP appeared to colocalize more extensively with an RGC marker, specifically an RNA-binding protein (RBPMS).

### 3.2. RNA-Seq Analysis Showed Few Genes Altered by 5 h Hypoglycemia

To decipher the genes involved in autophagosome formation, we performed laser capture microdissection (LCM), followed by RNA-Seq analysis of 6 GCL samples from 3 euglycemic (Eugly) and 3 hypoglycemic (Hypo) mice (Figure S1). To set up fixation conditions and determine the purity of isolated mRNA, we isolated both the GCL and outer nuclear layer (ONL) from two C57BL/6 mice using LCM, and tested the expression of G protein subunit alpha transducin 1 (GNAT1) and Thy-1 cell surface antigen (Thy-1) using qPCR (Figure S2). *THY1* was strongly expressed on GCL, with slight mRNA contamination by the ONL fraction in one sample (8% of GNAT1 expression); *GNAT1* was not detected in the second sample. Similarly, *GNAT1* was strongly expressed on ONL, with slight mRNA contamination in both GCL samples (2% and 6% of THY1 expression). This observation underscores the need for a good specific isolation process targeting GCL during LCM.

RNA-Seq analysis revealed very few changes in gene expression between Eugly and Hypo mice. Only three genes were differentially expressed, with an adjusted *p*-value of <0.05; many genes showed a high fold change with a low *p*-value, but not with a low adjusted *p*-value. Table 1A,B show the 10 genes that were up- and downregulated, respectively, under hypoglycemic conditions. Notably, these genes are not known to be involved in autophagy. Very few genes specific to autophagy appeared to be modified in the event of hypoglycemia; however, those overexpressed were not significant (they had a high adjusted *p*-value). We performed gene set enrichment analysis (GSEA) for genes positively and negatively involved in the control of autophagy (Figure S3). Despite there being no significant *p*-value, we observed an enrichment of positive genes in the hypoglycemic condition, whereas an enrichment of negative genes was observed in the euglycemic condition.

### 3.3. Hypoglycemia Induced Autophagy in RGCs by Impairing AL Formation

**Figure 4:**
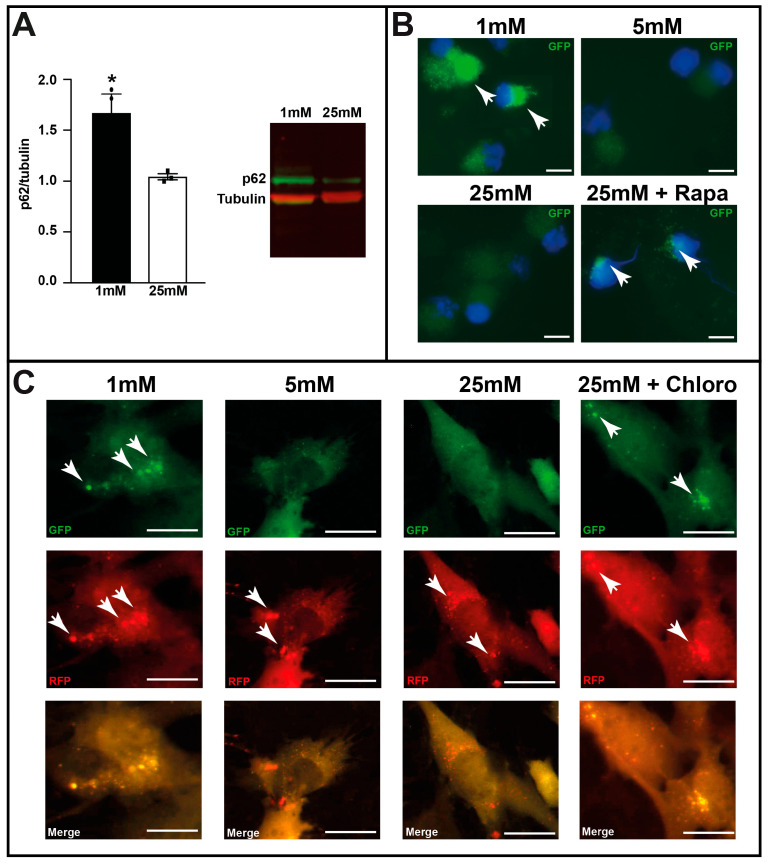
Low-glucose conditions induce autophagosome formation in isolated retinal ganglion cells (RGCs). (**A**) p62/SQSTM1 protein expression in isolated C57BL/6 RGCs cultured with 1 and 25 mM glucose for 48 h, assessed by Western Blot analysis. Results are expressed as mean ± SEM of 3 experiments. * *p* < 0.03. (**B**) GFP fluorescence (white arrows) of isolated GFP-LC3^tg/+^ RGCs cultured at various glucose concentrations (1, 5, and 25 mM); rapamycin (Rapa), an autophagic activator, was used as control. (**C**) GFP and RFP fluorescence (white arrows) of isolated C57BL/6 RGCs infected with mRFP-GFP-LC3 lentivirus and cultured at various glucose concentrations (1, 5, and 25 mM); chloroquine (Chloro), an inhibitor of autophagosome/lysosome fusion, was used as control. The images are representative of three different experiments. Scale bar 200 mm.

Owing to colocalization of autophagosomes (GFP staining) with RBPMS, we conducted several experiments on isolated RGCs (Figure S1). First, we isolated RGCs from C57BL/6 mice and cultured them at varying glucose concentrations. Western blot analysis showed a significant increase in SQSTM1/p62 (sequestosome 1) levels, suggesting a potential inhibition of autophagy or malfunction in the autophagic process (Figure 4A). Unfortunately, due to the limited protein lysate, it was technically not possible to detect LC3-II by Western blot analyses to confirm autophagosome accumulation. Therefore, we used GFP-LC3*^tg/+^* mice and isolated RGCs to culture them at varying glucose concentrations. Figure 4B clearly shows the accumulation of autophagosomes (GFP staining) with 1 mM glucose or in the presence of rapamycin, an autophagic activator, with 25 mM. No accumulation of GFP-LC3 puncta at baseline was observed (5 or 25 mM glucose alone). We then tested the fusion process of autophagosomes and lysosomes in C57BL/6 RGCs infected with the mRFP-GFP-LC3 lentivirus and cultured at diverse glucose concentrations in the presence or absence of chloroquine, an AL formation inhibitor. Normal autophagy occurred with 5 and 25 mM glucose, whereas 1 mM glucose and the presence of the autophagic inhibitor induced GFP fluorescence, suggesting a defect in the fusion process. qPCR analysis for lysosome-associated membrane protein 2 (Figure S4) showed a significant decrease in gene expression with 1 mM glucose compared to that with 5 and 25 mM glucose; protein expression of lysosome-associated membrane protein 2 could not be determined owing to a very low amount of sample after RGC isolation and culture. Both results suggest a defect in AL formation.

## 4. Discussion

We used the suggested guidelines to study the autophagy process in many, if not all, experiments [38]. To validate the use of the GFP-LC3 model [25] in retinal autophagy analyses, we intraocularly injected rapamycin into these mice; we chose to inject heterozygote mice because basal GFP fluorescence of homozygote GFP-LC3 mice was very strong, thereby masking the desired signal. Thereafter, we used heterozygote mice in all ex vivo and in vivo experiments. Our results indicate that rapamycin induced autophagosome formation in RGCs of both retinal explants (Figure 1) and mice (Figure 2). Therefore, this mouse model is appropriate for such studies. We also observed that RGCs were the major sites of autophagy induced by 5 h hypoglycemia. However, the autophagy process was blocked by a defect in AL formation, probably due to the downregulation of glucose-induced lysosome-associated membrane protein 2. Many studies demonstrated that autophagy has a protective effect, specifically on RGCs [19,20,23], whereas others found the opposite effect. One study showed that autophagy inhibition in glucose-deprived cortical neurons leads to less cell death [39]. In contrast, autophagy activation induces RGC death in a chronic hypertensive glaucoma model [24]. The effects of autophagy depend on the model used and the timing of analyses [40]. The main observation is the formation of autophagosomes, but it is unclear if complete fusion with lysosomes is achieved, especially in studies showing that autophagy activation induces cell death. These results are similar to the observation in this study, i.e., hypoglycemia induced autophagy; however, its protective effect was inhibited by a fusion process defect. Significant p62 overexpression and GFP fluorescence were observed when isolated RGCs were cultured under low-glucose conditions (Figure 4A) and infected with mRFP-GFP-LC3 (Figure 4C), respectively; both results support a defect in AL formation.

RNA-Seq analyses after LCM revealed that very few genes were significantly altered, when the adjusted *p*-value was used as a selection criterion. MyoD family inhibitor domain-containing 2 and taxilin-beta were upregulated, whereas cortexin 3 was downregulated. Since myoD family inhibitor domain-containing 2 is expressed in RGCs [41] and downregulated in streptozotocin-treated rats [42], no direct link between this protein and the RGC state was analyzed. Although no clear role for taxilin-beta in the retina has been described, this protein belongs to the syntaxin family, which is involved in intracellular vesicle trafficking [43]. Further analyses of this protein in the retina are needed to better understand its role, especially in autophagosomes or lysosomes. Cortexin 3 is considered a marker of intrinsically photosensitive RGCs [44]; it is downregulated after optic nerve crushing [45]. To date, these three proteins have not been linked to autophagy.

We were not able to highlight significant changes in the expression of key factors involved in autophagy, as expected; this may be due to the timing of analysis, which occurred shortly after the hyperinsulinemic/hypoglycemic clamp (4 h). However, we observed an enrichment of genes involved in the positive regulation of autophagy in isolated GCLs from mice that underwent a hypoglycemic condition, while genes involved in the negative regulation of autophagy were enriched in the euglycemic condition. Further analyses with different time points are necessary to detect autophagic markers. Other genes with expression modified during hypoglycemia, but not significantly so, are directly related to RGCs or have been seldom studied in the retina. There was an increase in the levels of SRY-box transcription factor 11 (SOX11), which has been implicated in RGC differentiation [46]. Some studies indicate that SOX11 promotes the survival of RGCs, specifically after optic nerve injury [47]; a recent study showed that SOX11 promotes axon regeneration, but is also responsible for killing other RGCs [48]. Further studies are needed to elucidate the role of hypoglycemia-induced SOX11 expression in RGCs, as well as its potential link with autophagy.

We recently showed that 5 h hypoglycemia in mice also induces ER stress (X-box binding protein 1 splicing) in many retinal cells, including the RGC layer [5]. As many reports describe a link between autophagy and ER stress (for review, see [11]), it will be interesting to further analyze whether ER stress can induce autophagy during hypoglycemia. In addition, a direct link between autophagy and retinal cell death has already been demonstrated in 661W cells [8], and we have preliminary results showing that modulation of GHS, a key player in apoptosis, affects the formation of autophagosomes.

This study has some limitations. We tested gene expression 4 h after 5 h hypoglycemia in mice. Autophagy analyses in a time- and dose-dependent manner will probably yield different and interesting data, mainly on autophagic markers. Furthermore, although we observed autophagy induced by hypoglycemia in two mouse lines (C57BL/6 and GFP-LC3), extrapolation of these results to patients with DR should always be conducted with a degree of caution. Furthermore, we used RGCs from newborn mice for ex vivo autophagy experiments because isolating adult RGCs is much more difficult and results in a low cell yield. Isolated RGCs from adult mice may produce different results. The metabolic state of a cell depends on the cell type and has a strong influence on autophagy. Here, we focused on RGCs due to in vivo results showing an accumulation of LC3 specifically in RGCs (Figure 3). However, we can assume that other retinal cells experience autophagy too, as was previously demonstrated in 661W photoreceptor cells [8]. The failure to detect significant changes in genes involved in autophagy may be due to issues related to RNA isolation following laser capture microdissection (LCM).

The impact of autophagy on neurodegeneration varies depending on the type of cell affected and the chronicity and severity of diabetic conditions, such as high or low glucose levels, oxidative and ER stress. It also depends on whether autophagic flux remains functional or becomes impaired. In the case of diabetic retinopathy, chronic and intense stress can cause the protective autophagic pathway to become saturated, inefficient, or defective, thus shifting its role from survival to promoting cellular dysfunction or death [14]. Furthermore, autophagy has been shown to play a neuroprotective role in RGCs, which are selectively lost in DR [49]. It’s suggested that a defect in the autophagic process, as observed in our hypoglycemic models or in low-glucose culture conditions, is part of the neurodegenerative process in RD. This defect could be a potential therapeutic target.

## 5. Conclusions

Herein, we have shown that 5 h hypoglycemia induced autophagosome formation, specifically in RGCs. Similarly to our findings in 661W photoreceptor cells [8], it appears that the autophagic process, more precisely the fusion between autophagosomes and lysosomes, is defective, resulting in apoptosis [4]. A better understanding of the fine equilibrium between ER stress, autophagy, and retinal cell death will provide insights into potential strategies to prevent diabetes-related complications such as DR.

## Figures and Tables

**Table 1:** RNA-Seq analysis after LCM in both hypoglycemic and euglycemic conditions. Panel A showed the 10 genes that were up- while panel B showed the 10 genes that were downregulated by hypoglycemia.

Gene ID	Gene Name	FC	*p* Value	*p* Value Adj	Description
(**A**)					
ENSMUSG00000090667	Gm765	6.5	8.17 × 10^−7^	0.013	MyoD family inhibitor domain containing-2
ENSMUSG00000039891	Txlnb	20.8	2.88 × 10^−6^	0.024	taxilin beta
ENSMUSG00000063632	Sox11	3.9	2.66 × 10^−5^	0.109	Transcription factor SOX-11
ENSMUSG00000084234	4933405O20Rik	90.0	7.30 × 10^−5^	0.182	Probable isocitrate dehydrogenase [NAD] gamma-2
ENSMUSG00000052374	Actn2	19.8	1.28 × 10^−4^	0.182	Alpha-actinin-2
ENSMUSG00000053228	Ceacam3	7.9	9.52 × 10^−5^	0.182	carcinoembryonic antigen-related cell adhesion molecule-3
ENSMUSG00000029862	Clcn1	5.5	1.44 × 10^−4^	0.182	Chloride channel protein-1
ENSMUSG00000030051	Aplf	5.5	1.36 × 10^−4^	0.182	Aprataxin and PNK-like factor
ENSMUSG00000051224	Tceanc	6.3	3.79 × 10^−4^	0.208	Transcription elongation factor A N-terminal and central domain-containing protein
ENSMUSG00000039480	Nt5dc1	4.7	3.29 × 10^−4^	0.208	5’-nucleotidase domain containing-1
**Gene ID**	**Gene Name**	**FC**	***p*** **Value**	***p*** **Value adj**	**Description**
(**B**)					
ENSMUSG00000069372	Ctxn3	−3.4	8.22 × 10^−6^	0.045	Cortexin-3
ENSMUSG00000030921	Trim30a	−14.4	1.06 × 10^−4^	0.182	Mus musculus tripartite motif-containing 30A
ENSMUSG00000063931	Pepd	−3.8	8.67 × 10^−5^	0.182	peptidase D
ENSMUSG00000000171	Sdhd	−3.3	9.49 × 10^−5^	0.182	succinate dehydrogenase complex
ENSMUSG00000097487	Ptges3l	−57.6	2.04 × 10^−4^	0.198	Putative protein PTGES3L
ENSMUSG00000020778	Ten1	−2.9	2.03 × 10^−4^	0.198	CST complex subunit TEN1
ENSMUSG00000029149	Krtcap3	−79.8	3.33 × 10^−4^	0.208	Keratinocyte-associated protein-3
ENSMUSG00000026090	2010300C02Rik	−50.5	2.63 × 10^−4^	0.208	RIKEN cDNA 2010300C02 gene
ENSMUSG00000047832	Cdca4	−36.6	3.51 × 10^−4^	0.208	Mus musculus cell division cycle associated-4
ENSMUSG00000041731	Pgm5	−6.0	3.31 × 10^−4^	0.208	Phosphoglucomutase-like protein-5

## Data Availability

The data that support the findings of this study are openly available on GEO, reference number (GSE292784).

## Supplementary Materials

The following supporting information can be downloaded at: https://www.mdpi.com/article/10.3390/cells14221774/s1, Figure S1: Diagrams explaining the different experiments; Figure S2: Determination of the purity of LCM isolated mRNA; Figure S3: Hypoglycemia in retinal ganglion-cell–layer (GCL) cells is associated with increased macroautophagy signaling. Figure S4: Lysosome-associated membrane protein 2 (Lamp2) is significantly decrease in retinal ganglion cell (RGC) cultured at low glucose.

## Abbreviations

AL	autolysosome
DR	Diabetic Retinopathy
ER	Endoplasmic Reticulum
Eugly	Euglycemic
GCL	Ganglion Cell Layer
GFAP	Glial Fibrillary Acidic Protein
GFP	Green Fluorescence Protein
GS	Glutamine Synthetase
GNAT1	G Protein Subunit Alpha Transducin 1
Hypo	Hypoglycemic
iBRB	Inner blood–retinal barrier (iBRB)
LCM	Laser Capture Microdissection
ONL	Outer Nuclear Layer
RGC	Retinal Ganglion Cells
RBPMS	RNA-Binding Protein
SOX11	SRY-Box Transcription Factor 11
Thy-1	Thy-1 Cell Surface Antigen
