# Supplementary material for: Autophagy Impairment in Retinal Ganglion Cells Following Hypoglycemia in Mice
Source: Cells. 2025 Nov 12;14(22):1774. doi: 10.3390/cells14221774 (PMC12651049; doi:10.3390/cells14221774)
Supplement: Supplementary file 1 [file cells-14-01774-s001.zip › cells-3896685-supplementary (1).pdf]

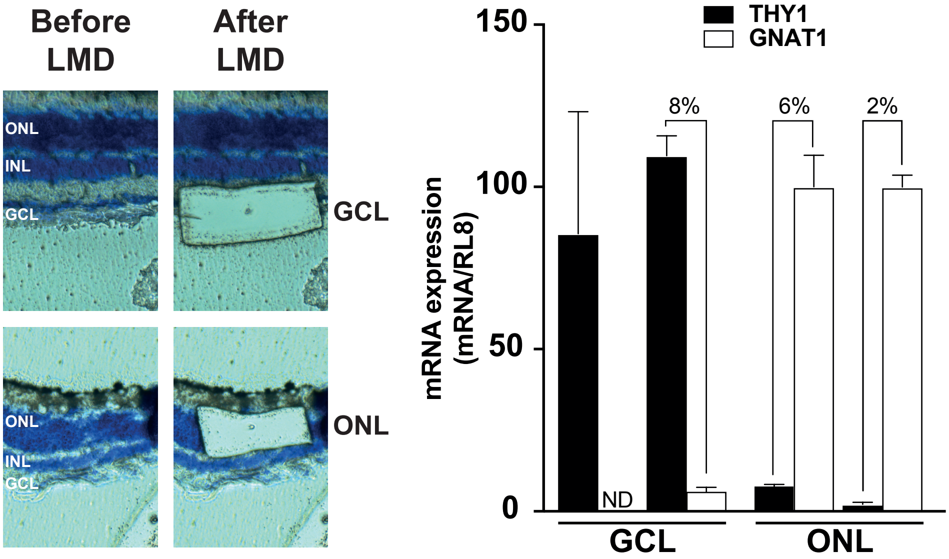

Figure S2: Determination of the purity of LCM isolated mRNA. We isolated both the ganglion cell layer (GCL) and outer nuclear layer (ONL) from two C57BL/6 mice using LCM, then we isolated mRNA and tested the expression of the G protein subunit alpha transducin 1 (GNAT1), which is a GCL marker and the Thy-1 cell surface antigen (Thy-1), which is an ONL marker using qPCR. We used the RL8 housekeeping gene expression to normalize. Percentage of contamination is assessed by setting the layer-specific marker at 100%.

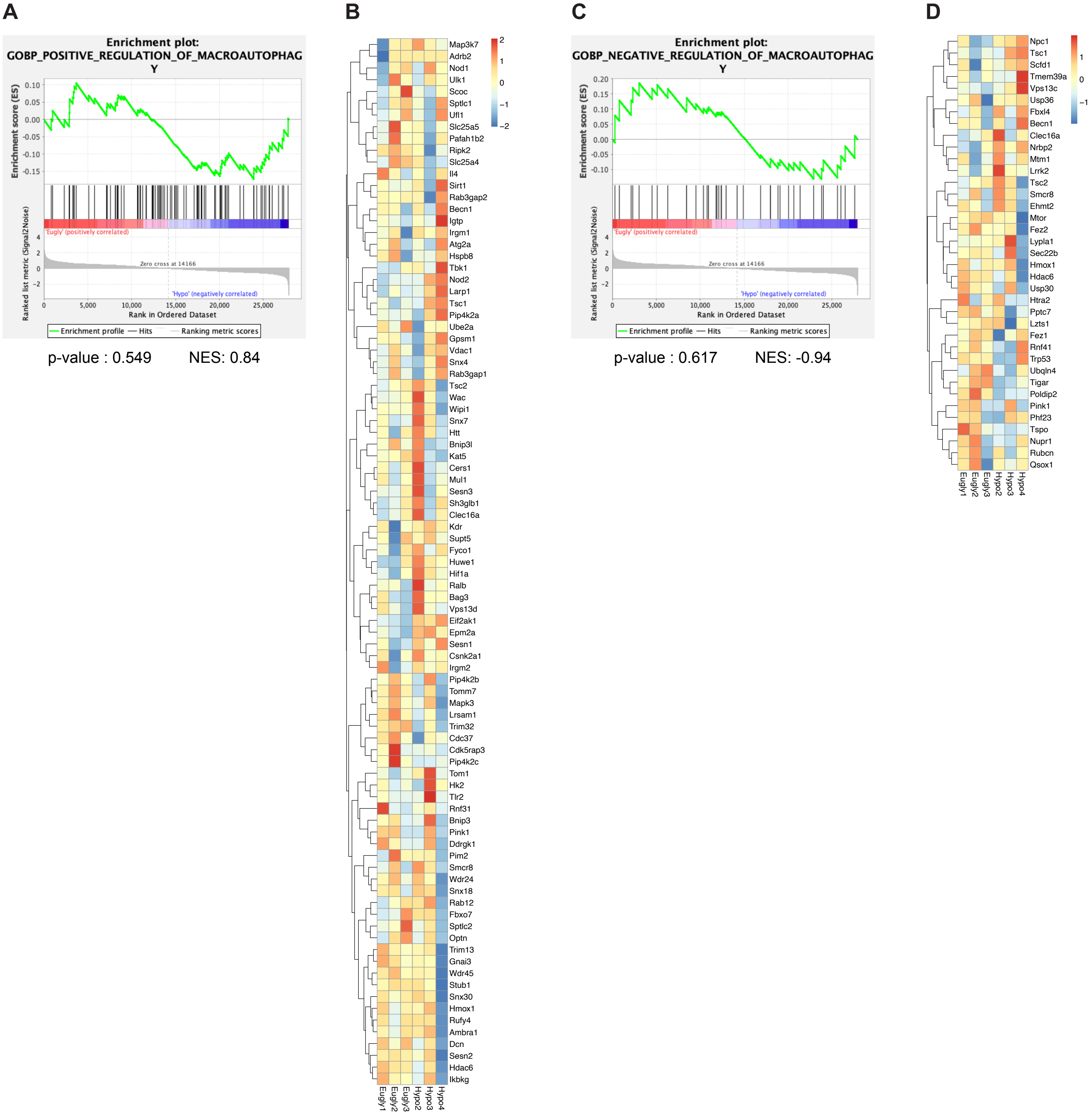

Figure S3: Hypoglycemia in retinal ganglion-cell–layer (GCL) cells is associated with increased macroautophagy signaling. (A, C) Gene Set Enrichment Analysis (GSEA) using Gene Ontology Biological Process (GO-BP) terms shows enrichment of « positive regulation of macroautophagy » in hypoglycemia (A) and enrichment of negative regulation of macroautophagy in euglycemia (C). Normalized enrichment scores (NES) and p-values are reported on the plots. (B, D) Heatmaps display the expression of genes from the corresponding GO-BP sets across samples. Values are scaled per gene (row Z-score).

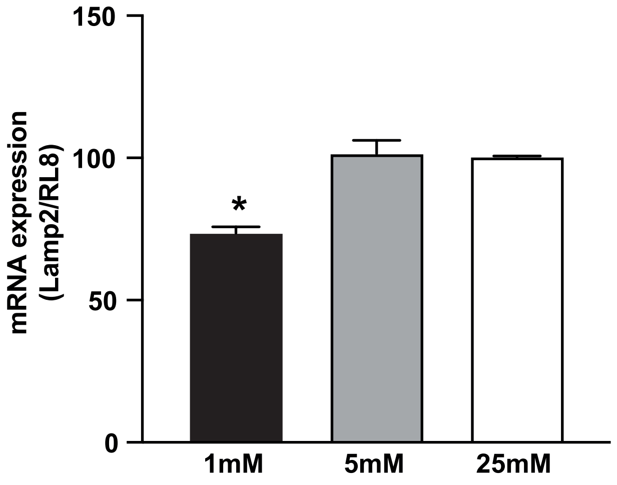

Figure S4: Lysosome-associated membrane protein 2 (Lamp2) is significantly decrease in retinal ganglion cell (RGC) cultured at low glucose. We cultured isolated C57BL/6 RGCs with 1, 5 and 25 mM glucose for 48 h, and then isolated mRNA to assess Lamp2 expression by qPCR analysis. We used the RL8 housekeeping gene expression to normalize. Results are expressed as mean ± SEM of 3 experiments. *$p < 0.05$
