# Supplementary figures and images for: Autophagy Impairment in Retinal Ganglion Cells Following Hypoglycemia in Mice
Source: Cells. 2025 Nov 12;14(22):1774. doi: 10.3390/cells14221774 (PMC12651049; doi:10.3390/cells14221774)

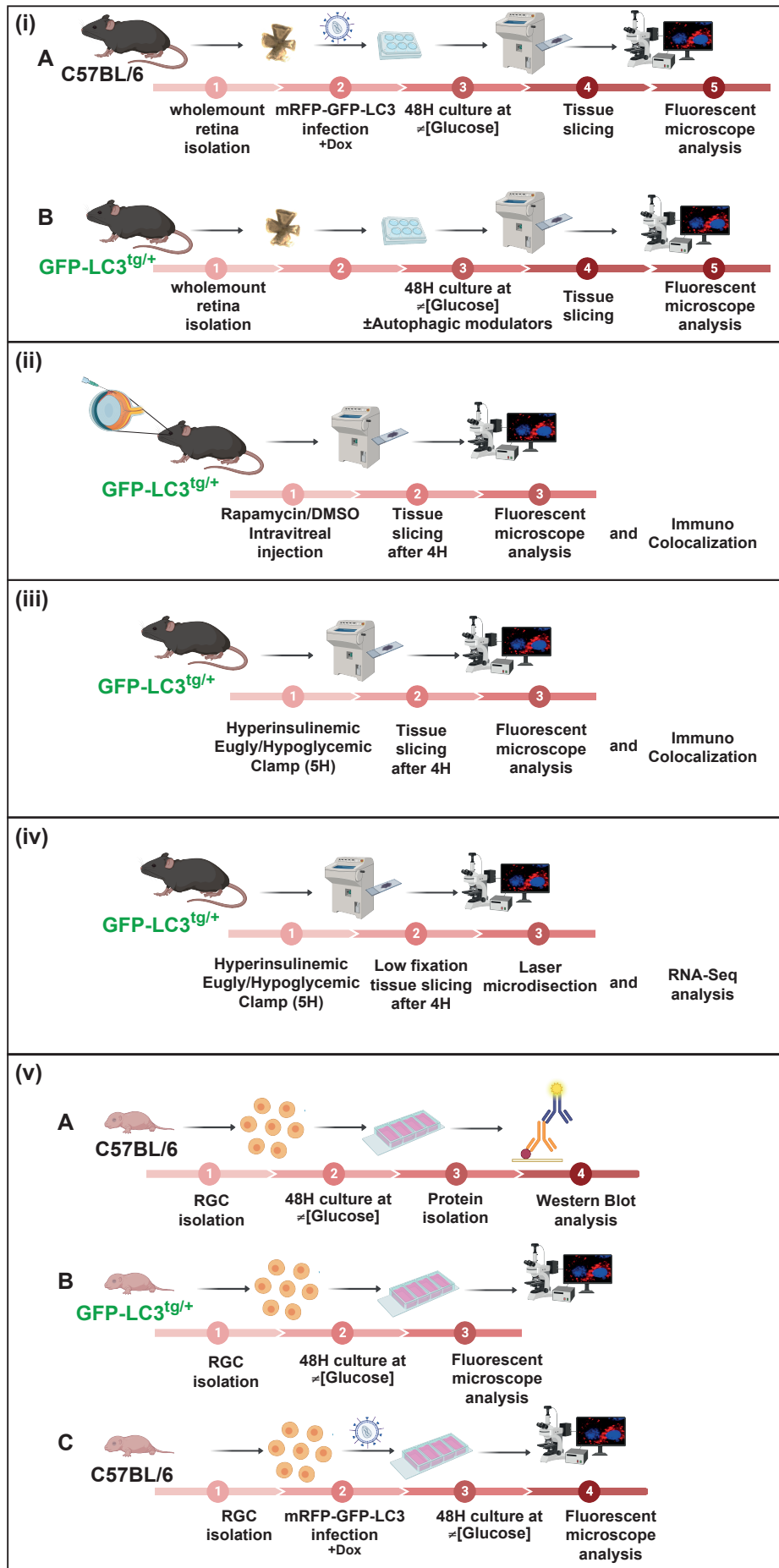

**Figure S1:** Diagrams explaining the different experiments based on the figures and table

Supplement: Supplementary file 1 [file cells-14-01774-s001.zip › cells-3896685 - 3rd proofreading-figure S1.pdf]
